# A rare presentation of type II Abernethy malformation and nephrotic syndrome: Case report and review

**DOI:** 10.1515/biol-2022-0086

**Published:** 2022-07-21

**Authors:** Xin Wu, Weizhong Gu, Yongzhi Lin, Lina Ye

**Affiliations:** Department of Pediatrics, Taizhou Central Hospital (Taizhou University Hospital), Taizhou 318001, China; Department of Pathology, The Children’s Hospital, Zhejiang University School of Medicine, National Clinical Research Center for Child Health, Hangzhou 310005, China; Department of Pediatrics Surgery, Taizhou Central Hospital (Taizhou University Hospital), Taizhou 318001, China; Department of General Surgery, Taizhou Central Hospital (Taizhou University Hospital), Taizhou 318001, China

**Keywords:** nephrotic syndrome, type II Abernethy malformation, congenital extrahepatic portosystemic shunt, liver biopsy

## Abstract

Type II Abernethy malformation is an extremely reported congenital extrahepatic portosystemic shunt in complication with nephrotic syndrome. We present the case of an 8-year-old boy who presented with symptoms of type II Abernethy malformation and nephrotic syndrome. This diagnosis of this type II Abernethy malformation was based on physical examination, blood tests, urinalysis, nephrotic and hepatic function tests, routine clinical lipid measurements, abdominal ultrasonography, and computed tomographic angiography. A kidney biopsy revealed the pathological features of nephrotic syndrome. This is the second reported patient diagnosed with type II Abernethy malformation and nephrotic syndrome. Captopril treatment was effective in improving the symptoms of this case. A patient with type II Abernethy malformation related to immune complex-mediated glomerular injury was effectively improved with medication. Type II Abernethy malformation is a causative factor of immune complex-mediated glomerular injury in nephrotic syndrome. Captopril treatment significantly improved the symptoms in this case.

## 1 Introduction

**Figure 1:**
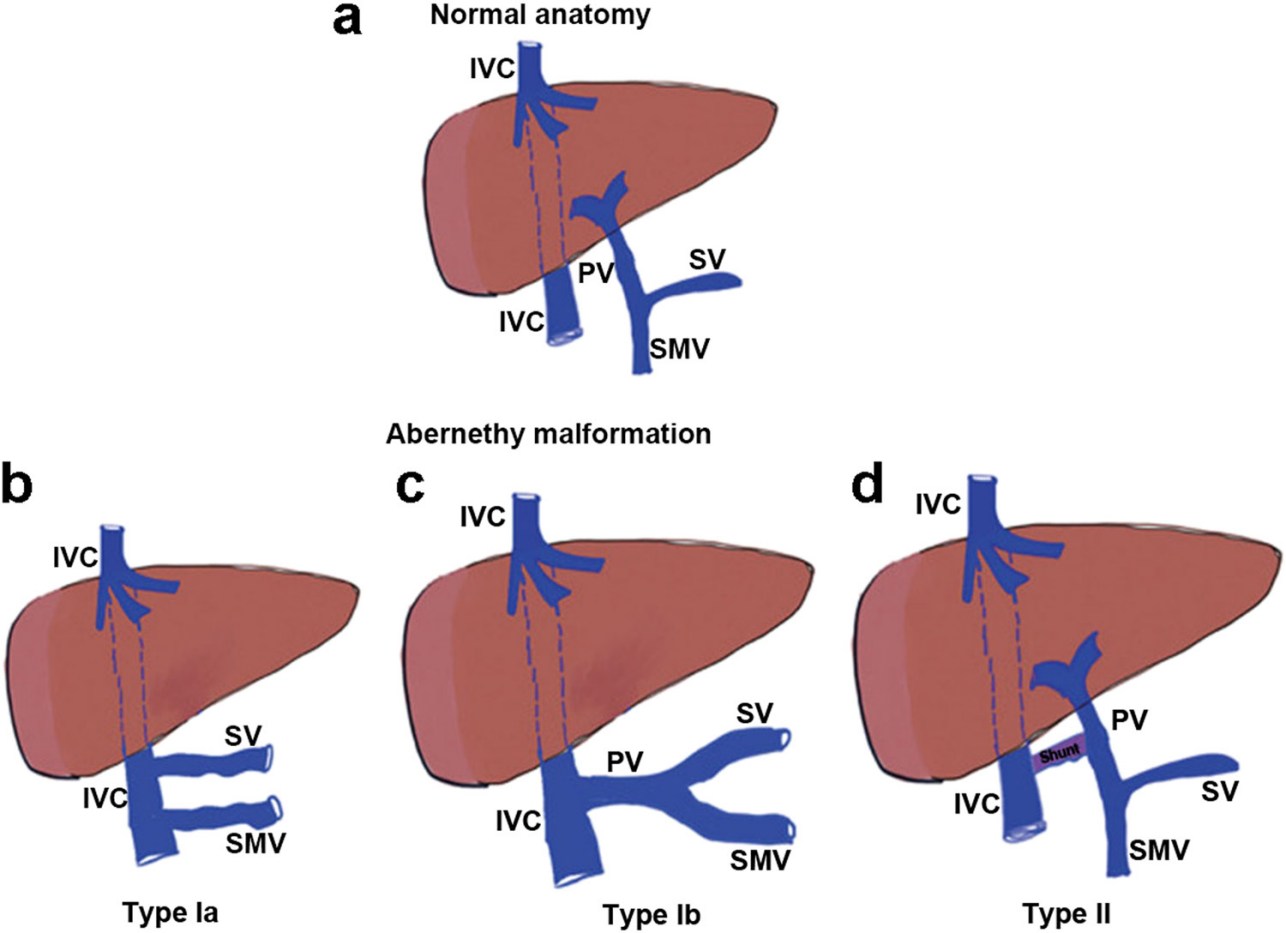
Hand-drawn diagrams suggesting congenital extrahepatic portosystemic shunts: (a) Graphic representation of normal portal flow; (b) Type Ia showing SV and SMV directly into the IVC; (c) Type Ib showing a presence of a connection between IVC and PV; (d) Type II representing a side-to-side communication between PV and IVC. SV, splenic veins; SMV, superior mesenteric vein; IVC, inferior vena cava; and PV, portal vein.

Abernethy malformation or congenital extrahepatic portosystemic shunt is an anomaly of the splanchnic venous flow that bypasses the liver and drains directly into the systemic circulation [1,2]. Abernethy first described Abernethy malformation in 1793 based on a postmortem examination [3]. After that, Morgan and Superina proposed the classification of Abernethy malformation into two types (Abernethy, types I–II) [4]. Type I Abernethy malformation shows direct draining of splenic vein (SV) and superior mesenteric vein (SMV) into the inferior vena cave (IVC) (Type Ia) or has a complete diversion of portal blood into IVC (Type Ib) (Figure 1, which was originally illuminated by Jian et al. [5]). In type II Abernethy malformation, a hypoplastic intrahepatic portal vein (PV) supplies partial venous blood to the liver. This rare congenital vascular malformation of the splanchnic venous system is due to the abnormal development of the umbilical vein in the embryo [1,6].

The clinical manifestation of Abernethy syndrome ranges from asymptomatic to presentations related to systemic or hepatic sequelae like pulmonary hypertension, hepatopulmonary syndrome, hepatic encephalopathy, liver nodules, or tumors (focal nodular hyperplasia, nodular regenerative hyperplasia, hepatocellular adenomas, and hepatocellular carcinomas) [2,7,8,9]. It is recognized that congenital portosystemic shunts are correlated with nephrotic syndrome [10]. Membranoproliferative glomerulonephritis (MPGN) is a specific histological form of glomerulonephritis with manifestations including diffuse mesangial hypercellularity, endocapillary proliferation, thickening of the capillary wall, lobulation of the glomerular tuft, and the split of the glomerular capillary wall. Type II Abernethy malformation was reported to be associated with immune complex-mediated MPGN. In this case report, we presented a patient diagnosed with type II Abernethy malformation and nephrotic syndrome. This patient suffered from hematuria and proteinuria related to increased renal vein pressure, which may be caused by portosystemic shunts.

### 1.1 Case presentation

An 8-year-old boy presented with scrotal swelling and both eyelids swelling for 2 days, and both lower extremities edema for 1 day. He had a history of hemorrhoids. Physical examination on admission revealed a normal body temperature (36.6^o^C), pulse rate (99/min), respiratory rate (24/min), and blood pressure (102/65 mmHg). The patient was conscious, mentally fine, and mildly anemic. No enlarged lymph nodes were palpated in the superficial lymph node region. The patient’s eyelids were edematous, and small red petechiae were visible on the left eyelid. The pharynx was not red, the respiratory sounds of both lungs were clear, dry, and wet rales were not heard, the heart sounds were strong and rhythmic, and no murmurs were heard. The abdomen was slightly distended, with no varices in the abdominal wall veins and no pressure pain or rebound pain in the whole abdomen. The liver was normal in shape, with no percussion pain in the kidney area. Percussion found alternating tympanic and turbid sounds in the abdomen. Both lower extremities showed sunken edema with a capillary filling time of less than 2 s. The patient received no relevant interventions. There is no hereditary family disease.

Results of the blood test suggested a white blood cell count of 6.3 × 10^9^/L (normal range: 4–12 × 10^9^/L), red blood cell count of 4.0 × 10^12^/L (normal range: 3.5–5.5 × 10^12^/L), neutrophil percentage of 43.0% (normal range: 50–70%), platelet count of 165 × 10^9^/L (normal range: 100–400 × 10^9^/L), and hemoglobin level of 119 g/L (normal range: 110–150 g/L). There was no abnormality in the erythrocyte sedimentation rate (11 mm/h). The level of high-sensitivity C-reactive protein was less than 0.2 mg/L (normal range: 0–10 mg/L). Urinary workup showed the number of urine erythrocytes was 178/HP (normal range: 0–3), the number of urine leukocytes was 22/HP (normal range: 0–4), and urine protein level was 2.0 g/L (3+) (normal range: negative). Renal function test results showed a urea level of 2.1 mmol/L (2.9–8.2), creatinine level of 17 μmol/L (62–115), and uric acid level of 218 μmol/L (208–428). Liver function and routine lipid determination results indicated a total bilirubin of 20.9 μmol/L (3.0–25.0), alanine aminotransferase activity of 20 U/L (9–50), aspartate transaminase activity of 56 U/L (15–40), globulin of 17.4 g/L (20–40), albumin/globulin ratio of 0.95 (1.20–2.40), total cholesterol of 7.14 mmol/L (3.10–5.70), triglyceride of 1.00 mmol/L (0.56–1.70), low-density lipoprotein cholesterol of 4.75 mmol/L (0.00–3.39), total protein of 33.9 g/L (65.0–85.0), and albumin of 16.5 g/L (40.0–55.0). Color Doppler ultrasonography showed no significant abnormalities in the epididymis and testes on both sides.

Abdominal ultrasonography indicated changes in hepatic echogenicity, absence of PV, and seroperitoneum. SV, SMV, and inferior mesenteric veins were dilated and tortuous. Extrahepatic portosystemic shunts were identified. Computed tomographic (CT) angiography showed the left and right branches of PV were quietly thin. SV and SMV converged at PV and drained directly into the IVC, which they communicated with the left external iliac vein. Aneurysmal dilatation of the pelvic veins was suggested. IVC, proximal hepatic veins, both renal veins, common iliac vein, and internal and external iliac veins were thickened. It was shown that the left renal vein was compressed.

**Figure 2:**
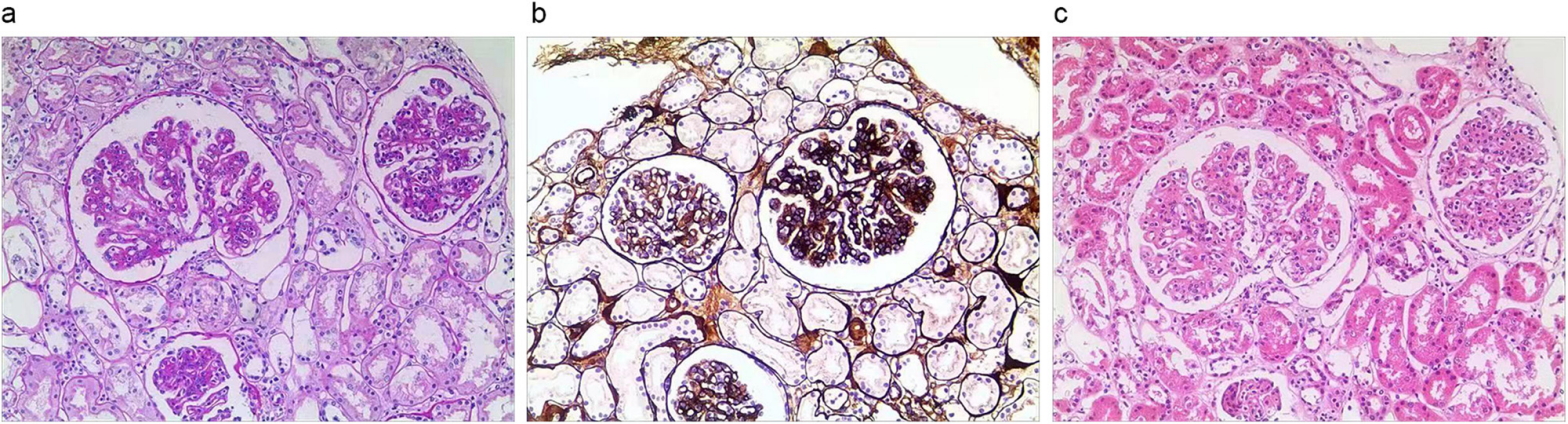
Representative photomicrographs of native biopsy: (a) HE, (b) Jones silver, and (c) PAS staining showed diffuse proliferation of mesangial cells and endocapillary hypercellularity.

A kidney biopsy was performed. Results from hematoxylin and eosin (HE), Jones silver, and periodic acid-Schiff (PAS) staining showed diffuse proliferation of mesangial cells and endocapillary hypercellularity (Figure 2). The light microscopy appearance was diffuse membranoproliferative patterns in mesangial and endothelial proliferation, accompanied by narrowed glomerular capillary and thickened capillary loops (Figure 2a and b). There was the focal segmental mesangial insertion in the glomerular basement membrane. The proliferation of mesangial cells and stroma was noticed. It was found in mesangial and subepithelial electron-dense deposits (Figure 2c). Immunofluorescence detected the glomerular deposition of IgG, IgA, IgM, C3 (0.283 g/L), C4 (0.037 g/L), and C1q. The pathological findings of the renal puncture suggested that electron-dense deposits, i.e., immune complex deposits, were seen in mesangial and paramesangial areas. The detachment of Sertoli cells was not observed. The pathology report suggested that the lesion is consistent with the characterization of mesangial proliferative glomerulonephritis. Systemic lupus erythematosus was ruled out because the patient was negative for antinuclear antibodies, dsDNA, nuclear acidic protien (Sm), and ribonucleoprotein. Moreover, there were no characteristic changes of systemic lupus erythematosus like hematoxylin bodies. Based on these results, the body was diagnosed with type II Abernethy malformation, nephritis with nephrotic syndrome, hypoproteinemia, hypocomplementemia, and hypofibrinogenemia.

Methylprednisolone (1 mg/kg/day) was administered to control inflammation for 2 weeks. Thereafter, the patient was administrated with prednisone (1 mg/time) in combination with tacrolimus (1 mg/time) twice per day for 2 weeks. The patient was treated with captopril (0.3–0.5 mg/kg/day) for 2 weeks and piperazine ferulate (1 tablet three times a day) for 2 weeks. Human serum albumin (200 mg/kg once) was administered to increase colloidal osmotic pressure. After the administration of the drug, the patient’s proteinuria improved, and the selling was significantly decreased. The patient is currently under follow-up in the Department of Pediatric Surgery and the Department of Nephrology.


**Informed consent:** Informed consent has been obtained from all individuals included in this study.
**Ethical approval:** The research related to human use has been complied with all the relevant national regulations, institutional policies and in accordance with the tenets of the Helsinki Declaration, and has been approved by the authors’ institutional review board or equivalent committee.

## 2 Discussion

The clinical manifestations of Abernethy malformation type II are quite diverse, ranging from invisible clinical signs to obvious severe complications such as gastrointestinal hemorrhage, liver tumors, hepatic encephalopathy, hepatopulmonary syndrome, and nephrotic syndrome [10,11,12,13,14,15]. The nephrotic syndrome is rarely reported with type II Abernethy malformation [10,15]. Reported here is the case of a patient with type II Abernethy malformation related to the immune complex-mediated glomerular injury that was successfully treated by medication therapy. In attempting to generalize the pathogenesis of this case, we reviewed previous reports about renal complications of type II Abernethy.

The symptoms of patients with type II Abernethy malformation vary from asymptomatic features to multi-organ failure [16,17]. Patients with type II Abernethy malformation presented signs of fatigue pitting edema, hyperammonemia, hypoxemia [18], anemia, hepatic encephalopathy [19], and varying degrees of hepatic impairment. Patients with a complication with complex congenital heart disease and a hepatopulmonary syndrome suggested interrupted IVC with a prominent azygos vein draining in the superior vena cava [16,17]. The combined type II Abernethy malformation and nephrotic syndrome showed decreased renal function, enlarged spleen, hypoplastic PV, portosystemic shunt, arachnoid cysts, and irregular deposition of IgG, IgA, IgM, C3, C1q, and C4 [15].

In the present case, laboratory tests revealed normal white blood cell count of 6.3 × 109/L (normal range: 4–12 × 109/L), red blood cell count of 4.0 × 1,012/L (normal range: 3.5–5.5 × 1,012/L), neutrophil percentage of 43.0% (normal range: 50–70%), platelet count of 165 × 109/L (normal range: 100–400 × 109/L), hemoglobin level of 119 g/L (normal range: 110–150 g/L), and high-sensitivity C-reactive protein less than 0.2 mg/L (normal range: 0–10 mg/L). There was no abnormality in the erythrocyte sedimentation rate (11 mm/h). Blood cultures were negative. In addition, the patient was improved without the application of antibiotics, which does not support the presence of n acute infection. The pathological findings of the renal puncture suggested that electron-dense deposits, i.e., immune complex deposits, were seen in mesangial and paramesangial areas. The detachment of Sertoli cells was not observed. The pathology report suggested that the lesion is consistent with the characterization of mesangial proliferative glomerulonephritis. The results from abdominal ultrasonography and CT angiography confirmed the diagnosis of type II Abernethy malformation. Kidney biopsy and immunofluorescence examinations indicated that this patient showed symptoms of nephrotic syndrome.

An important cause of type II Abernethy malformation is the abnormal development of the early embryo’s vascular system [13,20]. The presence of a portosystemic shunt has been revealed to be a causative factor for type II Abernethy malformation patients with multisystemic presentations (such as IgA glomerulonephritis, pulmonary hypertension, and multiple liver tumors) because its functional bypass decreases the clearance of immune complexes [10,15,21]. A postulated pathogenetic basis for IgA-predominant glomerulonephritis could be due to the increased bacteremia, vasoactive substances, and IgA-antigen complexes from the intestinal mucosa uncleared by the liver, causing deposition in the kidney after being filtered by the renal glomeruli [22,23]. Persistent chronic infection thus causes glomerulonephritis and kidney injury [22,23].

Abernethy malformation limits portal blood supply, which increases the risk of developing hepatic neoplasms [16]. As such, the early recognition of type II Abernethy malformation is particularly important, as well as other associated anomalies. Liver function examination revealed increased levels of alkaline phosphatase and aspartate transaminase [16]. In this case, a marked increase in liver enzyme levels occurred. The liver of type II malformation is still perfused due to partial shunting of blood through and side-to-side portosystemic shunt. Insufficient blood supply may lead to hepatocyte damage, which can explain the increase in liver enzyme levels. Non-invasive imaging technologies are extensively used for clinical diagnosis of type II Abernethy malformation, including ultrasound, CT, and magnetic resonance imaging (MRI). MRI and CT showed a lesion and multiple cysts in the liver, a vascular shunt between the left PV and IVC [16], interrupted IVC with a prominent azygos vein draining in the superior vena cava, polysplenia, and a bilobed liver connected to a dilated PV [17]. To confirm the congenital portosystemic shunt, a transjugular approach was used for hepatic vein and portal diagnostic venography [16]. Portal venous pressure measured by temporary balloon occlusion confirmed the patency of the right PV [16]. Cranial MRI was used to detect arachnoid cysts [15]. Kidney biopsy and immunofluorescence assays confirmed nephrotic syndromes like glomerulonephritis [15].

A treatment guideline for type II malformation has not been introduced at present. For patients without overt clinical symptoms, conservative treatment is prior [24]. Ligation of abnormal shunt vessels is considered for type II Abernethy malformation patients with excessive collateral circulation pressure, varicose veins, and hepatic encephalopathy [18,19]. Complete ligation of the portal-IVC fistula and narrowing PV and ICV improved the patient’s nonspecific abdominal pain and liver function while causing thrombus, which could be relieved by anticoagulation therapy (heparin infusion and rivaroxaban) [12]. For a type II Abernethy malformation patient with hepatocellular neoplasm, Arango et al. placed a 20-mm Amplatzer vascular plug II combined with coil embolization, which successively occluded the congenital portosystemic shunt, increased the tumor side, and improved liver function [16]. This interventional closure of the portosystemic shunt also shows the feasibility and safety for type II Abernethy malformation patients with complex congenital heart disease and hepatopulmonary syndrome [17]. Chick et al. innovatively used three-dimensional planning techniques to promote single-session Amplatzer atrial septal occlude device closure for type II Abernethy malformation, while vigilance is required for postoperative complications like splanchnic thrombosis and occlusion [25].

The presence of kidney disorder increases surgical risk [15]. Conservative medicine therapy with glucocorticoids and tacrolimus was considered because of kidney disorder, and this treatment achieved remission of the symptoms [15]. Although albuminuria was controlled by drug treatment in the short term, this long-term prognosis is not recommended [15]. Currently, there is no uniform protocol for the treatment of type II Abernethy malformation. Moreover, the patient had mild symptoms, so conservative medical treatment was chosen. In the present case, the urine protein level was decreased after captopril treatment. Captopril commonly induces the following side effects: hypotension, hyperkalemia, dry irritating cough, and angioneurotic edema. Hypotension often occurs when blood volume is insufficient. Captopril is not recommended for children with hyperkalemia. A dry irritating cough can be relieved by regulating the dosage used. Captopril reduces intra-glomerular hypertension and hyperperfusion by specifically regulating glomerular hemodynamics. Captopril decreases urinary protein and inhibits the accumulation of intracellular factors and extracellular matrix, thereby delaying the development of glomerulosclerosis, improving the prognosis, and protecting the kidney function. These effects are mainly attributed to its non-hemodynamic functions, including reduction of glomerular filtration pore size, prevention of small efferent artery constriction, and improvement of the glomerular basement membrane charge-selective barrier. Therefore, captopril has therapeutic advantages over other conservative drugs. However, persistent follow-up is recommended because of the high possibility of developing chronic renal failure, gastrointestinal bleeding, and malignant hepatic lesions.

In conclusion, the combined type II Abernethy malformation and the nephrotic syndrome were described in this case report. Type II Abernethy malformation is a causative factor for immune complex-mediated glomerular injury in nephrotic syndrome. Captopril treatment significantly improved the symptoms in this case. However, further close follow-up is necessary for observing the clinical effectiveness and disease progression.

## References

[1] Howard ER, Davenport M. Congenital extrahepatic portocaval shunts-the Abernethy malformation. J Pediatr Surg. 1997;32(3):494–7. 10.1016/s0022-3468(97)90614-x 9094026

[2] Baiges A, Turon F, Simón-Talero M, Tasayco S, Bueno J, Zekrini K, et al. Congenital extrahepatic portosystemic shunts (Abernethy Malformation): An international observational study. Hepatology. 2020;71(2):658–69. 10.1002/hep.30817 31211875

[3] Abernethy J. Account of two instances of uncommon formation in the viscera of the human body: From the philosophical transactions of the royal society of London. Med Facts Obs. 1797;7:100–8. PMC5111139 29106224

[4] Morgan G, Superina R. Congenital absence of the portal vein: two cases and a proposed classification system for portasystemic vascular anomalies. J Pediatr Surg. 1994;29(9):1239–41. 10.1016/0022-3468(94)90812-5 7807356

[5] Jain V, Sangdup T, Agarwala S, Bishoi AK, Chauhan S, Dhua A, et al. Abernethy malformation type 2: varied presentation, management and outcome. J Pediatr Surg. 2019;54(4):760–5. 10.1016/j.jpedsurg.2018.08.053 30262201

[6] Kumar P, Bhatia M, Garg A, Jain S, Kumar K. Abernethy malformation: A comprehensive review. Diagn Interv Radiol. 2022;28(1):21–8. 10.5152/dir.2021.20474. PMID: 34914605. PMC12278916 34914605

[7] Zhang XL, Duan XM, Wang FY, Zhang X, Sun Y, Ma N, et al. An infant with Abernethy malformation associated with heterotaxy and pulmonary hypertension. Chin Med J (Engl). 2017;130(18):2257–8. 10.4103/0366-6999.213978 PMC5598340 28875963

[8] Lemoine C, Nilsen A, Brandt K, Mohammad S, Melin-Aldana H, Superina R. Liver histopathology in patients with hepatic masses and the Abernethy malformation. J Pediatr Surg. 2019;54(2):266–71. 10.1016/j.jpedsurg.2018.10.083 30528201

[9] De Vito C, Tyraskis A, Davenport M, Thompson R, Heaton N, Quaglia A. Histopathology of livers in patients with congenital portosystemic shunts (Abernethy malformation): a case series of 22 patients. Virchows Arch. 2019;474(1):47–57. 10.1007/s00428-018-2464-4 PMC6323085 30357455

[10] Schaeffer DF, Laiq S, Jang HJ, John R, Adeyi OA. Abernethy malformation type II with nephrotic syndrome and other multisystemic presentation: an illustrative case for understanding pathogenesis of extrahepatic complication of congenital portosystemic shunt. Hum Pathol. 2013;44(3):432–7. 10.1016/j.humpath.2012.08.018 23245671

[11] Mesquita RD, Sousa M, Vilaverde F, Cardoso R. Abernethy malformation: beware in cases of unexplained hepatic encephalopathy in adults-case report and review of the relevant literature. BJR Case Rep. 2018;4(2):20170054. 10.1259/bjrcr.20170054 PMC6159114 30363163

[12] Zhou M, Zhang J, Luo L, Wang B, Zheng R, Li L, et al. Surgical ligation for the treatment of an unusual presentation of type II Abernethy malformation. Ann Vasc Surg. 2020;65:285.e1–5. 10.1016/j.avsg.2019.10.094 31705994

[13] Alvarez AE, Ribeiro AF, Hessel G, Baracat J, Ribeiro JD. Abernethy malformation: one of the etiologies of hepatopulmonary syndrome. Pediatr Pulmonol. 2002;34(5):391–4. 10.1002/ppul.10182 12357487

[14] Emre S, Arnon R, Cohen E, Morotti RA, Vaysman D, Shneider BL. Resolution of hepatopulmonary syndrome after auxiliary partial orthotopic liver transplantation in Abernethy malformation. A case report. Liver Transpl. 2007;13(12):1662–8. 10.1002/lt.21349 18044784

[15] He X, Zhu Y, Fu H, Feng C, Liu Z, Gu W, et al. Case Report: Membranoproliferative glomerulonephritis, a rare clinical manifestation of Abernethy malformation type II. Front Pediatr. 2021;9:647364. 10.3389/fped.2021.647364 PMC8010253 33816407

[16] Arango NP, Nishioka Y, Velasco J, Mahvash A, Mehran RJ, Wang LS, et al. Involution of hepatocellular neoplasm after embolization of a portosystemic vascular shunt in an adult with Abernethy Type II malformation. J Vasc Interv Radiol. 2021;32(9):1391–3. 10.1016/j.jvir.2021.05.026 PMC8415009 34462084

[17] Loureiro P, Georgiev S, Ewert P, Tanase D, Eicken A, Kammer B, et al. Successful percutaneous treatment with the Konar MF™-VSD Occluder in an infant with Abernethy syndrome-case report. Cardiovasc Diagn Ther. 2021;11(2):631–6. 10.21037/cdt-20-380 PMC8102267 33968640

[18] Zhang JS, Li L. Surgical ligation of a portosystemic shunt for the treatment of type II Abernethy malformation in 12 children. J Vasc Surg Venous Lymphat Disord. 2021;9(2):444–51. 10.1016/j.jvsv.2020.08.001 32791304

[19] Jiang C, Ye W, Liu C, Wu W, Li Y. Surgical ligation of portosystemic shunt to resolve severe hematuria and hemafecia caused by type II abernethy malformation. Ann Vasc Surg. 2015;29(5):1020.e11–6. 10.1016/j.avsg.2015.01.023 25770392

[20] Ghuman SS, Gupta S, Buxi TB, Rawat KS, Yadav A, Mehta N, et al. The Abernethy malformation-myriad imaging manifestations of a single entity. Indian J Radiol Imaging. 2016;26(3):364–72. 10.4103/0971-3026.190420 PMC5036336 27857464

[21] Raghuram KA, Bijulal S, Krishnamoorthy KM, Tharakan JA. Regression of pulmonary vascular disease after therapy of Abernethy malformation in visceral heterotaxy. Pediatr Cardiol. 2013;34(8):1882–5. 10.1007/s00246-012-0428-z 22843201

[22] Lhotta K. Beyond hepatorenal syndrome: glomerulonephritis in patients with liver disease. Semin Nephrol. 2002;22(4):302–8. 12118395

[23] Hemminger J, Arole V, Ayoub I, Brodsky SV, Nadasdy T, Satoskar AA. Acute glomerulonephritis with large confluent IgA-dominant deposits associated with liver cirrhosis. PLoS One. 2018;13(4):e0193274. 10.1371/journal.pone.0193274 PMC5892865 29634718

[24] Ding PX, Han XW, Liu C. Type II Abernethy malformation in a patient with primary budd-chiari syndrome. Ann Hepatol. 2019;18(1):246–9. 10.5604/01.3001.0012.7933 31113600

[25] Chick JFB, Reddy SN, Yu AC, Kelil T, Srinivasa RN, Cooper KJ, et al. Three-dimensional printing facilitates successful endovascular closure of a type II Abernethy malformation using an amplatzer atrial septal occluder device. Ann Vasc Surg. 2017;43:311.e15–23. 10.1016/j.avsg.2017.02.012 28502889

